# Leaf mass per area is independent of vein length per area: avoiding pitfalls when modelling phenotypic integration (reply to Blonder *et al.* 2014)

**DOI:** 10.1093/jxb/eru305

**Published:** 2014-08-12

**Authors:** Lawren Sack, Christine Scoffoni, Grace P. John, Hendrik Poorter, Chase M. Mason, Rodrigo Mendez-Alonzo, Lisa A. Donovan

**Affiliations:** ^1^Department of Ecology and Evolution, University of California Los Angeles, 621 Charles E. Young Drive South, Los Angeles, California 90095, USA; ^2^IBG-2 Plant Sciences, Forschungszentrum Jülich GmbH, D-52425 Jülich, Germany; ^3^Department of Plant Biology, University of Georgia, 2502 Miller Plant Sciences, Athens, Georgia 30602, USA

**Keywords:** Functional traits, leaf hydraulics, leaf mass per area, leaf nutrient concentrations, photosynthetic rate, vasculature, vein patterning.

## Abstract

It has been recently proposed that leaf vein length per area (***VLA***) is the major determinant of leaf mass per area (***LMA***), and would thereby determine other traits of the leaf economic spectrum (LES), such as photosynthetic rate per mass (*A*_mass_), nitrogen concentration per mass (*N*_mass_) and leaf lifespan (*LL*). In a previous paper we argued that this ‘vein origin’ hypothesis was supported only by a mathematical model with predestined outcomes, and that we found no support for the ‘vein origin’ hypothesis in our analyses of compiled data. In contrast to the ‘vein origin’ hypothesis, empirical evidence indicated that *VLA* and *LMA* are independent mechanistically, and *VLA* (among other vein traits) contributes to a higher photosynthetic rate per area (*A*_area_), which scales up to driving a higher *A*_mass_, all independently of *LMA*, *N*_mass_ and *LL*. In their reply to our paper, Blonder *et al.* (2014) raised questions about our analysis of their model, but did not address our main point, that the data did not support their hypothesis. In this paper we provide further analysis of an extended data set, which again robustly demonstrates the mechanistic independence of *LMA* from *VLA*, and thus does not support the ‘vein origin’ hypothesis. We also address the four specific points raised by Blonder *et al.* (2014) regarding our analyses. We additionally show how this debate provides critical guidance for improved modelling of LES traits and other networks of phenotypic traits that determine plant performance under contrasting environments.

## Highlight text

In a previous paper we clarified the roles of veins in determining leaf function. Here we contribute further data and address the concerns of Blonder *et al.* (2014), and provide guidance for phenotypic modelling.

## Introduction

Leaf economic spectrum (LES) relationships are a topic of fundamental interest. Fast-growing, resource-acquisitive species tend to have low leaf dry mass per area (*LMA*, which is equal to leaf thickness × leaf dry mass density), and high light-saturated rates of photosynthesis per mass (*A*_mass_), high nitrogen concentration per mass (*N*_mass_), and high respiration rate per mass (*R*_mass_), but shorter leaf lifespan (*LL*) relative to slow-growing, resource-conservative species (Small, 1972; Lambers and Poorter, 1992; Reich *et al.*, 1997; Wright *et al.*, 2004; Wright *et al.*, 2005; Donovan *et al.*, 2011; Heberling and Fridley, 2012; Edwards *et al.*, 2014; Reich, 2014). Blonder *et al*. (2011, 2013) proposed as the ‘origin’ of the LES that the leaf vein length per leaf area (*VLA*, also known as ‘vein density’) determines *LMA*, and thereby drives LES relationships both across diverse species as well as within species (the ‘vein origin’ hypothesis). Sack *et al.* (2013) argued that the simple mathematical model used by Blonder *et al*. to support their hypothesis was a circular argument leading to predestined outcomes. In our comprehensive analysis of previously published data for vein and LES traits, we found that *VLA* and *LMA* were mechanistically independent and uncorrelated in comparisons across large species sets, and that *VLA* influenced *A*_mass_ independently of *LMA*, *N*_mass_, and *LL*.

In a reply, Blonder *et al.* (2014) questioned some of our findings, i.e. that (1) their model was circular, (2) *VLA* and interveinal distance were manipulated unrealistically in their modelling, (3) their equations were not sensitive to vein traits, and (4) the published data did not support their assumptions and predicted correlations. In this paper we summarize the lack of support for the ‘vein origin’ hypothesis based on logic and data, and address the concerns raised by Blonder *et al*. Finally, we show how this debate provides useful guidance for future modelling of phenotypic integration.

## *LMA* is independent of *VLA*, and vein traits influence the LES independently of *LMA*

A one-trait-drives-all hypothesis, such as that *VLA* would determine LES traits and their inter-relationships, would be very appealing due to its simplicity, but attributing all that variation to one trait is an extraordinary proposition. This hypothesis was derived by Blonder *et al.* (2011) on the expectation that *VLA* and its negative correlate, interveinal distance (*IVD*), would determine *LMA* and leaf thickness (*LT*). A high *VLA* was hypothesized to drive a high *LMA* by contributing to the mass of the leaf, and a high *IVD* simultaneously to drive a high *LT* for optimal water flow, and thereby also drive a high *LMA*. These ideas are not mechanistically realistic because the leaf minor veins which determine *VLA* account for <5% of leaf volume and mass, and the relationship between *IVD* and *LT* is not fixed but highly variable across species (Sack *et al.*, 2013).

**Fig. 1.**
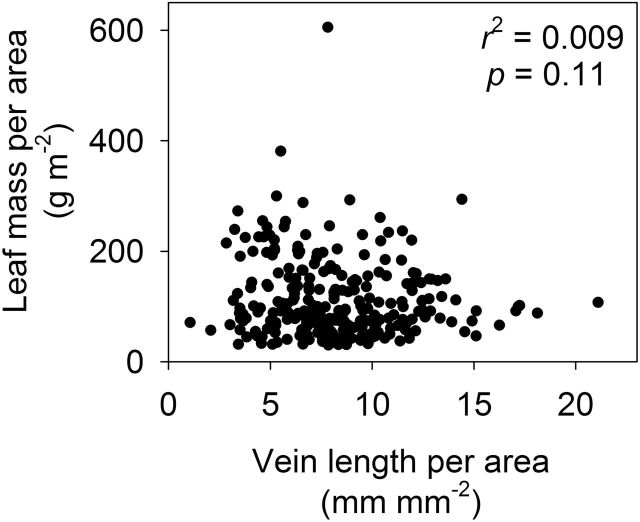
The independence of leaf mass per area (*LMA*) from vein length per leaf area (*VLA*) across phylogenetically diverse angiosperms. This is a replot of graph 3A of Sack *et al.* (2013), with additional data for 87 species of dicotyledons, for a total of 275 dicotyledonous species in 68 plant families. Additional data: six Hawaiian lobeliads, 29 Bolivian rainforest trees, and 52 species of Australian Proteaceae (data of Brodribb *et al.*, 2013; Jordan *et al.*, 2013; unpubl. data of L Sack, L Markesteijn, L Poorter, C Scoffoni, TJ Givnish, J Kunkle, R Montgomery, and M Rawls).

An extraordinary claim, such as the one-trait-drives-all ‘vein origin’ hypothesis, should require extraordinary evidence (Sagan, 1980). Instead, we found no empirical support. The clearest evidence against the ‘vein origin’ hypothesis is that *LMA* is independent of *VLA* across dicotyledons in a wide range of data sets, including within lineages of closely related species, and in a compiled database for 196 phylogenetically diverse species (Fig. 3A; Sack *et al.* 2013). The same is true after adding data that became recently available (total *n* = 275 species in 68 plant families; Fig. 1). Even within families, the data show that *VLA* and *LMA* are mechanistically independent. Among the nine families represented in our database by six or more species, *VLA* and *LMA* were correlated weakly within only Sapindaceae (log-transformed data; *R* ^2^ = 0.16; *P* < 0.05; *n* = 34), probably due to both variables being selected for greater values in species adapted to higher irradiance (i.e. a case of ‘concerted covergence’; Sack *et al.* 2013); for the other families no correlation was found (Campanulaceae, Fabaceae, Fagaceae, Myrtaceae, Plantaginaceae, Proteaceae, Rosaceae, and Violaceae; *R* ^2^ < 0.001–0.40; *P* = 0.18–0.97; *n* = 6–52).

A further, direct way to test the ‘vein origin’ hypothesis is by partial correlation analysis. Here one can determine whether the correlations among *A*_mass_, *LMA*, and *N*_mass_ are reduced or lost when *VLA* is partialed out—i.e. when the relationships are considered at a given *VLA*—as would be expected if *VLA* were the determinant of the relationships. However, when we applied that analysis to either the data set of Blonder *et al.* (2011) for 24 angiosperm species, or to the data set for the 114 angiosperm species in 48 families for which these traits were available in our compiled database of Sack *et al.* (2013), we found the opposite. In both data sets the inter-relationships among *A*_mass_, *LMA*, and *N*_mass_ remained significant (|*r*| = 0.49–0.78; *P* < 0.05) after accounting for *VLA* and indeed, the partial correlation coefficients did not differ from the raw correlation coefficients (paired *t*-test; *P* = 0.75–1.0; analyses applied for each data set with or without log-transformation of the data). These analyses demonstrate that *VLA* does not drive the LES trait relationships in the way that Blonder *et al*. proposed. Other leaf traits beyond vein traits play a well-known role in determining *LMA* and the LES relationships (see final section).

The ‘vein origin’ hypothesis is also not supported by a wider view of the plant kingdom that includes lineages in which leaves do not have minor veins. LES relationships are found in needle-leafed conifers, which only possess a single central vein (Reich *et al.*, 1998; Wright *et al.*, 2004); in ferns, which possess systems of few vein orders, and generally lack angiosperm-like minor veins (Karst and Lechowicz, 2007); in cycads, including species that lack any veins in their lamina other than the central midrib (Y. Zhang, K. Cao & L. Sack, unpubl. data); and even mosses, which lack any veins at all in their ‘leaves’ (Waite and Sack, 2010). Minor veins cannot be driving LES trait relationships in these lineages.

We thus concluded that the ‘vein origin’ hypothesis was not supported by critical evaluation of its assumptions or by the available data. Rather, we are of the opinion that in angiosperms vein traits influence LES traits and plant function in a different way—a higher *VLA* (among other vein traits) enables a higher photosynthetic rate per area (*A*_area_), which scales up to driving a higher *A*_mass_ and relative growth rate, independently of *LMA*, *N*_mass_, and *LL* (Sack *et al.*, 2013).

## Answering point 1: did the model predictions arise from circularity?

Blonder *et al.* (2011) argued for their ‘vein origin’ hypothesis based on a simple mathematical model, which we found to be circular, because its outcome was predetermined by inputs of other non-vein traits that drive the leaf economic spectrum traits (Sack *et al.* 2013). In their reply, Blonder *et al.* (2014) denied that their model was based on traits other than vein traits. Here, by stripping away the redundancies and revealing its core, we provide a new clarification of how their model predicts *LMA* and other LES variables from *LT*—a well-understood relationship, given that *LMA* = *LT* × leaf density (Witkowski and Lamont, 1991; Niinemets, 1999; Roderick *et al.*, 1999*b*).

**Fig. 2.**
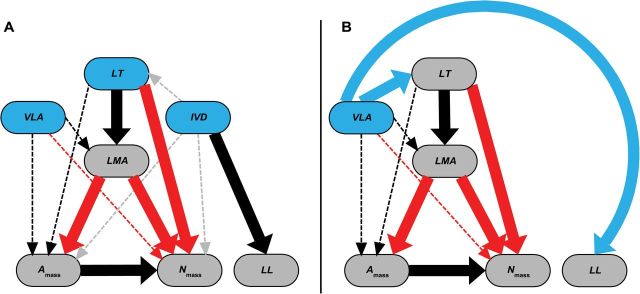
The structure of the ‘vein origin’ model of Blonder *et al.* (2011), based on eqns 4–7 (see Appendix 1), redrawn to highlight the influences of given variables (leaf mass per area, *LMA*; photosynthetic rate per mass, *A*_mass_; leaf nitrogen per mass, *N*_mass_; and leaf lifespan, *LL*; leaf thickness, *LT*; vein length per area, *VLA*; interveinal distance, *IVD*). This schema shows only the measured traits; other variables that were treated as constants are not included. Raw input traits are depicted in blue ovals; output traits are depicted in grey ovals (these are used as inputs for estimating other traits). The two panels show the contrasting implementation of equations for (A) prediction of leaf economics spectrum (LES) traits, and (B) for simulation of LES relationships. Black arrows represent positive influence according to eqns 4–7, red arrows negative influence. The thick arrows indicate the important drivers, and the thin dashed arrows represent negligible effects, according to sensitivity analyses (Table 2) and randomization analyses (Sack *et al.* 2013); the grey dotted arrows linking *IVD* to most variables represent drivers apparent in the equations that cancel out when the equations were rewritten as eqns 4a, 5a and 7a. When the model was implemented for prediction (A), *LT*, *VLA*, and *IVD* were inputted, and the estimates of LES traits were driven by measurements of *LT*, which resulted in weak relationships among the estimated LES variables and weak correlations between estimated and observed values for LES traits, independently of vein trait inputs, which have negligible effects in these equations. When the model was implemented for simulation (B), *VLA* was used to directly determine *LT* and *LL*, not reflecting a real mechanism, indicated by blue arrows. Thus, the input of *VLA* drove all output traits in the simulation, forcing the predetermined outcome in which *VLA* appears to drive LES trait relationships.

Blonder *et al.* (2011) proposed that the LES traits *LMA*, *N*_mass_, *A*_mass_, and *LL* are determined by four equations (eqns 4–7; Blonder *et al.*, 2011; see also Appendix 1) that require inputs of *LT*, other LES traits, and constant values for many other traits that are highly variable across species in reality. Two vein traits are also inputted, *VLA* and *IVD*. However, these vein traits either cancel out if the equations are re-written more simply, or have negligible effect on the predictions because they are multiplied by very small numbers in the equations (see eqns 4a, 6a, and 7a; Appendix 1). For clarity, we have re-drawn Fig. 1 of Blonder *et al.* (2014), without the constants, and using our symbols, after correction of inaccuracies in their depictions of their Eqns 4–7 (Fig. 2 of this paper).

They implemented these equations in two ways (*LES trait prediction* and *LES relationship simulation*; Fig. 2A and 2B in this paper). For *LES trait prediction* (Fig. 2A in this paper) they applied the four equations to data for 24 species, and found weak correlations between predicted and observed LES trait values (*R* ^2^ = 0.10–0.35; Fig. S3 in Blonder *et al.* 2011). Our sensitivity analyses demonstrated that this weak predictive power was caused by the use of *LT* and LES traits as inputs in eqns 4, 6, and 7, with negligible influence of vein traits *per se* (see ‘Addressing point 3’, below). Randomizing the *VLA* had no impact on the predictions of LES traits using these equations (Sack *et al.*, 2013).

The second modelling approach they used was *LES relationship simulation* (Fig. 3 in both Blonder *et al.*, 2011 and Blonder *et al.*, 2014). Here, realistic data for *VLA* were inputted into the equations, which apparently drove LES relationships among the output variables *LMA*, *N*_mass_, *A*_mass_, and *LL*. However, if they had run simulations using their model just as described above for prediction, they would have found a negligible sensitivity to *VLA*. For their simulations, they used a different equation (eqn 4b or 4c, Appendix 1) in which *VLA* was used to directly determine *LT*, by assuming a constant ratio of *IVD* to *LT* (where the ratio of *IVD* to *LT* was defined as ‘*k*_o_’ and made equal to 1), and a constant leaf tissue mass density for all species. Then, Blonder *et al*. used this simulated *LT* to directly determine *LMA* and the other LES variables (Fig. 2B in this paper). Thus, they ‘wrote in’ a direct dependency of *LT*, *LMA*, and the other LES traits on *VLA* (eqns 4b and 4c, Appendix 1). The predicted LES relationships fall in the centre of the global data set for LES traits because the constants in the equations were chosen for that purpose. Of course, one might consider this as simply a theoretical exercise to represent scaling up a scenario where *VLA* truly did drive *LT* and *LMA* perfectly, according to those assumptions. However, this does not reflect reality: *IVD* (or *VLA*) and *LT* are only correlated in some species sets and decoupled in others, and weakly correlated across diverse species (Table 3 of Sack *et al.*, 2013), and hence *k*_o_ is not optimized at a single value but varies widely across angiosperm species (with values varying more than 10-fold, ranging from 0.85 to 9.9 across 85 angiosperm species; Zwieniecki and Boyce, 2014). Even within families, *VLA* and *LT* are not generally tightly optimized; *VLA* and *LT* were negatively correlated in only two of the six families in our database represented by six or more species, Proteaceae and Violaceae (log-transformed data; *R* ^2^ = 0.42–0.59; *P* <0.05; *n* = 46 and 8, respectively); the other four families showed no significant relationships (Campanulaceae, Fabaceae, Plantaginaceae, and Sapindaceae; *R* ^2^ = 0.002–0.26; *P* = 0.30–0.80; *n* = 6–31). Blonder *et al.* (2014) have recognized that the linkage of *IVD* (or *VLA*) with *LT* can be weak, but did not address the implications of this for their model. Additionally, leaf tissue mass density is not constant as they assumed, but highly variable across species (Witkowski and Lamont, 1991; Niinemets, 1999), and for most groups of species is a more important determinant of *LMA* and the other variables of the LES than leaf thickness (Poorter *et al.* 2009). Consequently, as shown above, *LMA* is independent of *VLA* across diverse species (Fig. 1).

Attempting to demonstrate that *VLA* drives the LES by generating values for *LT* and *LMA* directly as a function of *VLA*, and then using these values to generate other LES traits, and then showing the resulting variables to be inter-related, without a realistic basis, is arguing in a circle, also known as ‘begging the question’. Such an argument implicitly asserts in one of the premises of an argument what is desired as the outcome, and is not valid evidence in support of a hypothesis (Damer, 2001). Any other variable (e.g. sunlight or herbivory) could be written in this way as a driver for *LT* and *LMA* in eqn 4, and by this circularity would have been ‘supported’ as the origin of the LES. Such modelling proves only its predetermined conclusion.

The same issues exist in the extended model of Blonder *et al.* (2013), which additionally includes flexible, unspecified parameters, and thus cannot be tested against data (Appendix 3 of Sack *et al.*, 2013).

## Answering point 2: did the model treat *IVD* and *VLA* as correlated or uncorrelated?

It is a well-known fact that *IVD* and *VLA* are inversely related by geometry, as first shown by Jane Philpott (1953) and reviewed in Sack *et al.* (2013). Blonder *et al.* (2014) agreed with us, but disputed our statement that they had ever considered them to be independent. However, in deriving their equations they had considered *IVD* and *VLA* to be independent enough that they could both be positively correlated with *LMA* and *LL* (their eqns 4–7; Fig. 1 of Sack *et al.* 2013 and of Blonder *et al.* 2014). Further, in their predictive modelling, Blonder *et al.* (2011) used these variables as independent inputs (eqns 4–7), contrary to what they depicted in their Fig. 1. On the other hand, in their simulation modelling, as discussed in ‘*Answering Point 1*’, they did indeed consider these traits as perfectly correlated, and translated *VLA* into *IVD* and then into *LT* values (eqns 4b and 4c). Thus, they treated the *IVD* and *VLA* as independent or non-independent as a matter of convenience—whichever led to a desired outcome. We advocate that models based on leaf vein traits incorporate the intrinsic correlations for *VLA* and *IVD* at each step, rather than treating them as fully independent or perfectly correlated depending on context.

## Answering point 3: what do the sensitivity and randomization analyses show?

Blonder *et al.* (2014) questioned whether our sensitivity analysis was mathematically correct and relevant. In our paper we compared the raw partial derivatives for their eqns 4–7, which tests the numerical impact of shifts in the input variables, at their actual values, units, and scales, on the output variable. That type of sensitivity analysis is well established in mathematical modelling (e.g. Hamby, 1994; Fasso and Perri, 2002). The analysis showed that the LES traits had the dominant influence on the outputs of eqns 4, 5, and 7, with a negligible role for *VLA*. Blonder *et al.* (2014) suggested that it would be better to use a ‘relative’ partial derivative sensitivity analysis, which tests the sensitivity of the output variable to a given proportional change in given input variables (Hamby, 1994; Fasso and Perri, 2002). That analysis actually results in the same conclusion: the equations are insensitive to vein traits (Table 1). In the example they presented, they estimated for their Eqn 4a that the sensitivity of *LMA* to a 10% shift in *VLA* was a third of its sensitivity to a 10% shift in *LT*, and they considered these influences to be ‘nearly equivalent’. However, using a more accurate across-species mean *LT* of 300 µm rather than their 100 µm (Poorter *et al.* 2009), the sensitivity of *LMA* to a 10% shift in *VLA* was a tenth of that for a 10% shift in *LT* (Table 1). This extremely low sensitivity to *VLA* in eqn 4a was due to its multiplication by the square of a small number, i.e. the inputted radius of a minor vein (*r*_v_) of 20 µm reported from a previous study of wheat leaves (Altus *et al.*, 1985) and assumed by Blonder *et al*. to be constant across all species, and independent of *VLA*. Those assumptions are not valid, as recent work has shown that across dicotyledonous species, leaves with higher *VLA* tend to have narrower minor veins (Feild and Brodribb, 2013): across 111 species, *r*_v_ scaled with *VLA* ^–0.6^. If that scaling relationship is inserted into eqn 4a to increase its accuracy, the left-hand term containing *VLA* becomes even more negligible (with *VLA* now raised to a –0.2 power), and the equation becomes altogether insensitive to *VLA*. In plain terms, the mass and volume of the minor veins is very small, due to their having such narrow diameters—and if the fact that the diameter is indeed negatively related to *VLA* is taken on board, the contribution of the veins to leaf volume and mass becomes even more negligible. The equations for *N*_mass_ and *A*_mass_ (eqns 5 and 7) likewise show them to be determined by *LT* and *LMA* and negligibly affected by *VLA* (Table 1). All these analyses prove that their equations have outputs negligibly driven by vein traits, and determine LES traits from *LT* and other LES traits.

**Table 1.** Results of a ‘relative’ partial derivative sensitivity analysis of eqns 4, 5, and 7 of Blonder *et al.* (2011)

		Sensitivity^a^ to input variable (i.e. shift in output variable due to a 10% shift in input variable)			
Output variable	Eqn	*VLA*	*LT*	*LMA*	*A*_mass_
*LMA* (g m^–2^)	4	0.880	**9.00** ^b^		
*A*_mass_ (nmol g^–1^ s^–1^)	5	0.249	0.213	**–37.5** ^b^	
*N*_mass_ (%)	7	–0.00120	**–0.0287** ^b^	**–0.0273** ^b^	**0.115** ^b^

^a^ Sensitivity = the partial derivative of the output variable with respect to each input variable (∂*y*/∂*x*) × a mean value for the input variable × 10%. This gives the influence on the output variable (in the given units) of a 10% shift in the input variable. ^b^ Values in bold italics are those which have >10 x the influence on the output variable than *VLA*. Mean trait values used: *VLA*, 10mm mm^–2^; LT, 300 µm; *LMA*, 110g m^–2^; light-saturated *A*_mass_, 115 nmol g^–1^ s^–1^; foliar *N*_mass_, 2% (based on the database of Sack *et al.*, 2013). For partial derivative formulae, see Appendix 2 of Sack *et al.* (2013).

The same result was found in a randomization analysis: eqns 4, 6, and 7 produced the same predicted values for *LMA*, *N*_mass_, and *A*_mass_ from the data set of Blonder *et al*. even when the *VLA* values were randomized (Fig. 6 in Sack *et al.*, 2013). In an effort to challenge this demonstration, Blonder *et al.* (2014) claimed to have repeated our analysis but found a different result (their Fig. 4 and attached R script). That analysis, however, did not use their eqns 4–7 as given, or as used for their original prediction. Rather, they used eqn 4b, in which *VLA* acts as a direct proxy for *LT*, and then randomized *VLA*, thus effectively randomizing *LT*; this obviously has a major impact on predictions of *LMA* and other LES traits. As we showed, when simply applying eqns 4, 5, and 7 without such manipulation, and randomizing *VLA* in a real or realistic data set, one finds negligible influence of *VLA* on LES trait estimation. We conclude that it is critical to conduct detailed sensitivity analyses and/or appropriately designed randomization analyses to fully understand a model prior to its publication.

## Answering point 4: what do the data show?

Our examination of vein trait correlations was based on 14 studies and in considering all traits, included data for over 350 species from 88 families. It is the most systematic and comprehensive database to date and did not support the assumptions or predictions of the ‘vein origin’ hypothesis, as summarized in Table 3 and Figs 1 and 3 of Sack *et al.* (2013). Rather than confront this fact, Blonder *et al.* (2014) have instead claimed that all the data supported their hypothesis, and six times misreported our findings and those from other published papers. These statements are listed and corrected in Table 2. We contend that data must be respected as the only means to support or (in this case) to falsify a hypothesis such as the ‘vein origin’ hypothesis.

**Table 2.** Misreporting of data by Blonder *et al.* (2014) to claim support for their ‘vein origin’ hypothesis

Topic	Reporting by Blonder *et al.* (2014)	Actual finding or statement in Sack *et al.* (2013) or other literature
Correlation of *LMA* and *VLA*	‘Our model proposes that *VLA* should be correlated with *LMA.* In the data cited by Sack *et al*. ... three of three data sets support the *LMA*– *VLA* linkage [their Table 3.2]’	Table 3, row 2 reported that only one data set of six tested for *LMA* vs *VLA* showed the positive correlation predicted by Blonder *et al.* (2011). As stated in the text that single data set was for species of *Acer* adapted across a light gradient, and thus the trend was probably due to co-selection of both traits during adaptation to contrasting irradiances. Fig. 3a of Sack *et al.* (2012) showed *LMA* and *VLA* are independent when data are compiled across many species; see Fig. 1 of this paper for the updated version, now for 276 species in 68 plant families. Table 3, row 2 of Sack *et al.* (2012) also reported that neither of two data sets showed the positive correlation of *LMA* vs *IVD* predicted by Blonder *et al.* (2011).
Correlation of *N*_mass_ and *VLA*	‘Our model proposes that *VLA* should be correlated with … *N*_mass_. and no data are presented for the *N*_mass_–*VLA* linkage except our 2011 results, which support predictions.’	Such a direct relationship was not proposed by Blonder *et al.* (2011); they hypothesized that *N*_mass_ was determined by eqn 7, which in fact predicts a negligible, but negative influence of *VLA* (Table 2 of this paper). The *VLA*–*N*_mass_ relationship was not significant across species- means in Blonder *et al.* (2011) (*r* = 0.27; *P* = 0.20). That relationship was also not supported in our compiled database (*n* = 162 species for this test; *r* = 0.08; *P* = 0.32).
Correlation of *LL* and *VLA*	‘Our model proposes that *VLA* should be correlated with *LL.* In the data cited by Sack *et al.* … three of three data sets support the *LL*–*VLA* linkage [their Table 3.4]’	Blonder *et al.* (2011) predicted that *LL* would be correlated with *VLA* due to their shared positive relationship with *LMA*. Table 3, row 4 of Sack *et al.* (2012) showed that in three of three data sets *LL* tended to be weakly correlated negatively with *VLA* across species, but in all three of three data sets this trend was not driven by the mechanism they proposed, since it occurs independently of *LMA* (i.e. the trend exists even when *LMA* is partialled out). As also described in Table 3, row 4 of Sack *et al.* (2012), this trend probably arises due to co-selection of both traits during adaptation to contrasting environments. In the one data set tested, the trend disappeared for *Helianthus* when mean annual precipitation was partialled out.
Correlation of *A*_mass_ with *VLA*	‘Our model proposes that *VLA* should be correlated with *A*_mass_ … In the data cited by Sack *et al.* … one of one data set supports the *A*_mass_–*VLA* linkage [their Fig. 8]’	Such a direct relationship was not proposed by Blonder *et al.*, 2011; they hypothesized that *A*_mass_ was determined by Eqn 6, which was negligibly sensitive to *VLA* (Table 2 of this paper). The *VLA*–*A*_mass_ relationship was not significant across species- means in Blonder *et al.* (2011) (*r* = 0.34; *P* = 0.13). This relationship was shown for the first time to our knowledge in a large compiled data set for 119 species by Sack *et al.* (2012). Contrary to the vein origin hypothesis, this relationship arose independently of *LMA*, because *VLA* drove *A*_area_ (Fig. 8 of Sack *et al.*, 2012).
Contribution of minor veins to leaf volume	‘the volume contribution of minor veins does play an important role in high-*VLA* leaves (Feild and Brodribb, 2013)’	Feild and Brodribb (2013) showed that in high *VLA* leaves, the minor vein diameter was lower and thus the minor vein volume per leaf area (= *VLA* × π × minor vein radius^2^) was lower: ‘Many of the most densely veined angiosperm leaves known bound the lower limits of leaf cost, with low leaf mass per area’.
Overall support for their model	‘Sack *et al.* (2013) examined the theoretical basis and empirical evidence for the Blonder *et al.* (2011) venation model and found limited support.’ ‘Sack *et al.* (2013) … feel that empirical support for the proposed correlations is weak.’ ‘At this point, multiple lines of evidence at both the intra- and interspecific scale are consistent with the main predictions of the Blonder *et al.* models.’	We found no support at all for the ‘vein origin’ hypothesis and clearly stated this in the Abstract and throughout the 2013 paper.

## Outlook: the need for mechanistic and fallacy-free models for phenotypic integration

This debate highlights important principles for modelling of traits in integrated phenotypes.

First, a more sophisticated approach than applying correlative models to simple leaf traits in an attempt to elucidate the LES as Blonder *et al.* (2014) recommend, is to develop an explanatory model based on the underlying traits known to be important. Anatomical and compositional determinants have been described for *LMA* (e.g. Garnier and Laurent, 1994; Pyankov *et al.*, 1999; Roderick *et al.*, 1999*a*; Poorter *et al.*, 2009; John *et al.*, 2013; Villar *et al.*, 2013), for *N*_mass_ (e.g. Villar *et al.*, 2006; Funk *et al.*, 2013), and for rates of photosynthesis (e.g. Terashima *et al.*, 2011; Tosens *et al.*, 2012; Tomás *et al.*, 2013) and respiration (Buckley and Adams, 2011; Tcherkez *et al.*, 2012), and leaf lifespan (Onoda *et al*. 2011). These direct determinants should form the basis for a mechanistic model of the LES. The influence of other plant traits that also influence the LES should be considered, such as branching architecture and growth form, which can influence *LL* and its relationship with *LMA* (Funk and Cornwell, 2013; Edwards *et al.*, 2014).

Indeed, we advocate considering the wider set of traits that influence a given function, rather than excluding them or treating them as constants. The ‘flux-traits’ hypothesis we presented for the influence of vein traits on plant function was synthesized from the literature and makes testable predictions for a much larger set of leaf traits, and their influence on whole plant function; parts of this framework have been applied mathematically in several previous studies (e.g. McKown *et al.*, 2010; Osborne and Sack, 2012; Flexas *et al.*, 2013). Blonder *et al.* (2014) claimed that this framework is ‘overly parameter rich’, but we presented a network of traits known to be involved, and it was not exhaustive. We acknowledge a role for simplified models. However, ignoring information of leaf anatomy and function in favour of a model at odds with what is known, as they did, is unlikely to move forward our understanding of plant function or ecological processes.

Likewise, mechanistic models for the underlying basis and function of traits should be based on the state of the art understanding of processes. Blonder *et al.* (2011) developed their equations (particularly those for *N*_mass_ and *A*_mass_) using incorrect assumptions on the physics and biology of gas exchange and the hydraulics of water transport. For example, they assumed that the leaf hydraulic conductance is negligible rather than strongly limiting to transpiration rate and *A*_area_ (Sack and Holbrook, 2006; Brodribb *et al.*, 2007; Brodribb *et al.*, 2010), that *N*_mass_ is causally determined by *A*_mass_ rather than the other way around (Fig. 2), that minor veins contribute strongly to leaf volume and mass, and many more. They assigned constant values to traits that vary enormously across species, such as vein diameters, mass density of lamina, and stomatal density. Such an approach is risky as it can lead to a ‘house of cards’ situation where the model can lose both mechanistic realism and predictiveness. Our ‘flux-traits’ hypothesis for the influence of vein traits on plant function is explicitly mechanistic, supported, and testable by measurements of anatomy and physiological processes.

It is also important for modelling to be conducted at the right scale. Thus, contrary to what Blonder *et al*. have argued, emerging understanding of the genetic basis of vein traits in *Arabidopsis* does not automatically support the ‘vein origin’ hypothesis, which made no predictions for any genetic linkages. Recent studies have indicated correlations across genotypes of vein traits with mesophyll and epidermal cell size, lamina thickness, and other traits, all of which should be associated with LES traits, according to their common genetic and developmental basis (Perez-Perez *et al.*, 2011; Sack and Scoffoni, 2013). At a higher scale, studies of the linkage of venation traits with plant performance, species-distributions, community assembly, and their relationships to climate benefit from data sets of simpler traits such as *VLA* and *LMA* for many species (Sack & Scoffoni, 2013). For maximum progress, such studies should be informed by sound knowledge and fallacy-free models for trait-based mechanisms; e.g. the ‘flux-traits’ hypothesis predicts that these traits will have mechanistically independent impacts on whole plant relative growth rate (Sack *et al.*, 2013).

Finally, we advocate that in mathematical modelling the desired output variable is in no way inputted or ‘written in’ to the model. This can be avoided by conducting sensitivity analyses to identify the major drivers of the output variables.

Developing new models with improved realism will lead to increased predictive power, especially if these avoid a single-trait focus, take on board known underlying variables and mechanisms, consider traits at the right scale, avoid circularity, and apply sensitivity analyses. Models of such quality are increasingly essential for understanding phenotypic structure/function trait networks, discoveries of the underlying basis for key physiological rates, and for predicting plant performance and larger scale ecological patterns under contrasting environments.

## Funding

This work was supported by National Science Foundation Grant 1147292.

## Appendix 1. Equations presented by Blonder *et al.* (2011) as their ‘vein origin’ hypothesis, and used for predictive and simulation modelling

The equations are presented using symbols as in Table 1 of Sack *et al.* (2013) with equation numbers as presented in that paper and Blonder *et al.* (2011).

### Prediction of LMA

$$LMA=\pi r_{v}^{2}VLA\left(\rho_{v}-\rho_{L}\right)+\frac{2\rho_{L}IVD}{k_{o}}$$ (4)

where *r*_v_, ρ_v_, and ρ_L_ are respectively the vein bundle radius, the mass density of veins, and the mass density of lamina; *k*_o_ is the ratio of interveinal distance to half the leaf thickness: *k*_o_ = *IVD* / (0.5 × *LT*), where *LT* is leaf thickness. Eqn 4 can be rewritten as

$$LMA=\pi r_{v}^{2}VLA\left(\rho_{v}-\rho_{L}\right)+(LT\times \rho_{L})$$ (4a)

This is the equation that Blonder *et al.* (2011) used to predict *LMA* values from observed data for *VLA* and *LT*. This was not necessarily clear, because they stated that they used measured *IVD* values and measured *k*_o_ values in eqn 4; the *IVD* thus cancelled out and effectively eqn 4a was used. Sensitivity analyses showed that, of its inputs that are not constants, this equation is driven by *LT*, rather than *VLA*, because the constants in the left-hand term render the influence of *VLA* very small (Table 1).

For their simulation modelling, Blonder *et al*. modified their approach. They replaced *IVD* in the right-hand term of eqn 4 with *VLA*, by estimating *IVD* as 1/*VLA* or 2/*VLA* (their reticulate and non-reticulate network simulations, respectively). This allowed input of *VLA* into the right-hand term of eqn 4, to which the equation is sensitive. Additionally, they fixed *k*_o_ as a constant value of 1, although this is an unrealistic assumption (see main text), and forces *VLA* to drive *LMA* in this equation. Thus, according to this formulation,

$$LMA=\pi r_{v}^{2}VLA\left(\rho_{v}-\rho_{L}\right)+\frac{2\times \rho_{L}}{VLA}$$ (4b)

or,

$$LMA=\pi r_{v}^{2}VLA\left(\rho_{v}-\rho_{L}\right)+\frac{4\times \rho_{L}}{VLA}$$ (4c)

Thus, through this manipulation, 1/*VLA* or 2/*VLA* was inserted into the right-hand term of eqn 4, such that it acts as a direct proxy for *LT* in eqn 4a, and thus drives *LMA*.

### Prediction of LL

$$LL=k_{\text{1}}IVD$$ (5)

where *k*_1_ was set to a constant value chosen to allow predictions of realistic *LL* values.

### Prediction of A_*mass*_

$$A_{\text{mass}}=\frac{c_{o}\left(1-h\right)WUE}{\pi r_{v}^{2}VLA\left(\rho_{v}-\rho_{L}\right)+\frac{2\rho_{L}IVD}{k_{o}}}\frac{1}{\frac{1}{\frac{\pi D\times VLA}{2log\frac{IVD}{k_{o}r_{v}}}}+\frac{1}{D\frac{a_{s}n_{s}}{t_{s}+\sqrt{a_{s}/\pi }}}}$$ (6)

where *c*_o_, *h*, *WUE*, *D*, *a*_s_, *n*_s_, and *t*_s_ are respectively the saturation vapour concentration of water in air, relative humidity, water use efficiency, the diffusion constant of water in air, stomatal pore area, stomatal density, and stomatal pore thickness.

Eqn 6 can be rewritten as

$$A_{\text{mass}}=\frac{WUE}{LMA}\frac{c_{o}\left(1-h\right)}{\frac{1}{\frac{\pi D\times VLA}{2log\frac{0.5LT}{r_{v}}}}+\frac{1}{D\frac{a_{s}n_{s}}{t_{s}+\sqrt{a_{s}/\pi }}}}$$ (6a)

This is the equation that Blonder *et al.* (2011) used to predict *A*_mass_ values from observed data for *VLA*, *LMA*, and *LT*. This was not necessarily clear, because they stated that they used measured *IVD* values and measured *k*_o_ values in eqn 6; the *IVD* thus cancelled out and effectively eqn 6a was used. Sensitivity analyses showed that, of its inputs that are not constants, this equation is driven by *LMA*, rather than *VLA*, because constants in the equation render the influence of *VLA* very small (Table 1).

For their simulation modelling, Blonder *et al*. modified their approach. They replaced *IVD* in eqn 6 with *VLA*, by estimating *IVD* as 1/*VLA* or 2/*VLA* (their reticulate and non-reticulate network simulations, respectively). This allowed an additional direct input of *VLA* into equation 6. Additionally, they fixed *k*_o_ as a constant value of 1, although this is an unrealistic assumption (see main text). In simulations, *A*_mass_ was driven by *LMA* which in turn was driven by *LT*, which was defined as a direct proxy of *VLA* (see above).

### Prediction of N_*mass*_

$$N_{\text{mass}}=k_{2}A_{\text{mass}}+\frac{k_{3}}{k_{0}}\frac{2\times IVD-k_{0}\pi r_{v}^{2}\times VLA}{LMA}$$ (7)

For *r*_v_, ρ^v^, ρ_L_, *a*_s_, *n*_s_, and *t*_s_, Blonder *et al*. used constants based on the literature, though values in fact vary widely among species. The *k*_2_ and *k*_3_ were set to constant values chosen to allow predictions of realistic *N*_mass_ values.

Eqn 7 can be rewritten as

$$N_{\text{mass}}=k_{2}A_{\text{mass}}+\frac{k_{3}(LT-\pi r_{v}^{2}\times VLA)}{LMA}$$ (7a)

This is the equation that Blonder *et al.* (2011) used to predict *N*_mass_ values from observed data for *VLA*, *LMA*, *LT*, and *A*_mass_. This was not necessarily clear, because they stated that they used measured *IVD* values and measured *k*_o_ values in eqn 7; the *IVD* thus cancelled out and effectively eqn 7a was used. Sensitivity analyses showed that, of its inputs that are not constants, this equation is driven by *A*_mass_, rather than *VLA*, because constants in the equation render the influence of the other variables very small (Table 1).

For their simulation modelling, Blonder *et al*. modified their approach. They replaced *IVD* in eqn 7 with *VLA*, by estimating *IVD* as 1/*VLA* or 2/*VLA* (their reticulate and non-reticulate network simulations, respectively). This allowed an additional direct input of *VLA* into eqn 7. Additionally, they fixed *k*_o_ as a constant value of 1, although this is an unrealistic assumption (see main text). In simulations, *N*_mass_ was driven by *A*_mass_ which in turn was driven by *LMA*, which was in turn driven by *LT*, which was defined as a direct proxy of *VLA* (see above).

## References

[CIT0001] Altus DP Canny MJ Blackman DR 1985 Water pathways in wheat leaves. II. Water-conducting capacities and vessel diameters of different vein types, and the behaviour of the integrated vein network. Australian Journal of Plant Physiology 12, 183–199.

[CIT0002] Blonder B Violle C Bentley LP Enquist BJ 2011 Venation networks and the origin of the leaf economics spectrum. Ecology Letters 14, 91–100. 21073643 10.1111/j.1461-0248.2010.01554.x

[CIT0003] Blonder B Violle C Bentley LP Enquist BJ 2014 Inclusion of vein traits improves predictive power for the leaf economic spectrum: a response to Sack *et al*. (2013). Journal of Experimental Botany 65, 5109–5114. 24723403 10.1093/jxb/eru143 PMC4400534

[CIT0004] Blonder B Violle C Enquist BJ 2013 Assessing the causes and scales of the leaf economics spectrum using venation networks in *Populus tremuloides* . Journal of Ecology 101, 981–989.

[CIT0005] Brodribb TJ Feild TS Jordan GJ 2007 Leaf maximum photosynthetic rate and venation are linked by hydraulics. Plant Physiology 144, 1890–1898. 17556506 10.1104/pp.107.101352 PMC1949879

[CIT0006] Brodribb TJ Feild TS Sack L 2010 Viewing leaf structure and evolution from a hydraulic perspective. Functional Plant Biology 37, 488–498.

[CIT0007] Brodribb TJ Jordan GJ Carpenter RJ 2013 Unified changes in cell size permit coordinated leaf evolution. New Phytologist 199, 559–570. 23647069 10.1111/nph.12300

[CIT0008] Buckley TN Adams MA 2011 An analytical model of non-photorespiratory CO_2_ release in the light and dark in leaves of C_3_ species based on stoichiometric flux balance. Plant, Cell and Environment 34, 89–112. 10.1111/j.1365-3040.2010.02228.x 21039609

[CIT0009] Damer TE 2001 Attacking faulty reasoning: a practical guide to fallacy-free arguments . Belmont, California: Wadsworth/Thomson.

[CIT0010] Donovan LA Maherali H Caruso CM Huber H de Kroon H 2011 The evolution of the worldwide leaf economics spectrum. Trends in Ecology & Evolution 26, 88–95. 21196061 10.1016/j.tree.2010.11.011

[CIT0011] Edwards EJ Chatelet DS Sack L Donoghue MJ 2014 Leaf life span and the leaf economic spectrum in the context of whole plant architecture. Journal of Ecology 102, 328–336.

[CIT0012] Fasso A Perri PF 2002 Sensitivity analysis. In: El-Shaarawi AH Piegorsch WW, eds. Encyclopedia of environmetrics . Chichester: Wiley, 1968–1982.

[CIT0013] Feild TS Brodribb TJ 2013 Hydraulic tuning of vein cell microstructure in the evolution of angiosperm venation networks. New Phytologist 199, 720–726. 23668223 10.1111/nph.12311

[CIT0014] Flexas J Scoffoni C Gago J Sack L 2013 Leaf mesophyll conductance and leaf hydraulic conductance: an introduction to their measurement and coordination. Journal of Experimental Botany 64, 3965–3981. 24123453 10.1093/jxb/ert319

[CIT0015] Funk JL Cornwell WK 2013 Leaf traits within communities: context may affect the mapping of traits to function. Ecology 94, 1893–1897. 24279259 10.1890/12-1602.1

[CIT0016] Funk JL Glenwinkel LA Sack L 2013 Differential allocation to photosynthetic and non-photosynthetic nitrogen fractions among native and invasive species. PLoS One 8. 10.1371/journal.pone.0064502 PMC3659119 23700483

[CIT0017] Garnier E Laurent G 1994 Leaf anatomy, specific mass and water content in congeneric annual and perennial grass species. New Phytologist 128, 725–736.

[CIT0018] Hamby DM 1994 A review of techniques for parameter sensitivity analysis of environmental models. Environmental Monitoring and Assessment 32, 135–154. 24214086 10.1007/BF00547132

[CIT0019] Heberling JM Fridley JD 2012 Biogeographic constraints on the world-wide leaf economics spectrum. Global Ecology and Biogeography 21, 1137–1146.

[CIT0020] John GP Scoffoni C Sack L 2013 Allometry of cells and tissues within leaves. American Journal of Botany 100, 1936–1948. 24070860 10.3732/ajb.1200608

[CIT0021] Jordan GJ Brodribb TJ Blackman CJ Weston PH 2013 Climate drives vein anatomy in Proteaceae. American Journal of Botany 100, 1483–1493. 23935111 10.3732/ajb.1200471

[CIT0022] Karst AL Lechowicz MJ 2007 Are correlations among foliar traits in ferns consistent with those in the seed plants? New Phytologist 173, 306–312. 17204077 10.1111/j.1469-8137.2006.01914.x

[CIT0023] Lambers H Poorter H 1992 Inherent variation in growth rate between higher plants: a search for physiological causes and ecological consequences. Advances in Ecological Research 23, 187–261.

[CIT0024] McKown AD Cochard H Sack L 2010 Decoding leaf hydraulics with a spatially explicit model: principles of venation architecture and implications for its evolution. American Naturalist 175, 447–460. 10.1086/650721 20178410

[CIT0025] Niinemets U 1999 Components of leaf dry mass per area – thickness and density – alter leaf photosynthetic capacity in reverse directions in woody plants. New Phytologist 144, 35–47.

[CIT0026] Osborne CP Sack L 2012 Evolution of C_4_ plants: a new hypothesis for an interaction of CO_2_ and water relations mediated by plant hydraulics. Philosophical Transactions of the Royal Society B: Biological Sciences 367, 583–600. 10.1098/rstb.2011.0261 PMC3248710 22232769

[CIT0027] Perez-Perez JM Rubio-Diaz S Dhondt S Hernandez-Romero D Sanchez-Soriano J Beemster GTS Rosa Ponce M Micol JL 2011 Whole organ, venation and epidermal cell morphological variations are correlated in the leaves of *Arabidopsis* mutants. Plant, Cell and Environment 34, 2200–2211. 10.1111/j.1365-3040.2011.02415.x 21883289

[CIT0028] Philpott J 1953 A blade tissue study of leaves of forty-seven species of *Ficus* . Botanical Gazette 115, 15–35.

[CIT0029] Poorter H Niinemets U Poorter L Wright IJ Villar R 2009 Causes and consequences of variation in leaf mass per area (LMA): a meta-analysis. New Phytologist 182, 565–588. 19434804 10.1111/j.1469-8137.2009.02830.x

[CIT0030] Pyankov VI Kondratchuk AV Shipley B 1999 Leaf structure and specific leaf mass: the alpine desert plants of the Eastern Pamirs, Tadjikistan. New Phytologist 143, 131–142.

[CIT0031] Reich PB 2014 The world-wide ‘fast-slow’ plant economics spectrum: a traits manifesto. Journal of Ecology 102, 275–301.

[CIT0032] Reich PB Ellsworth DS Walters MB 1998 Leaf structure (specific leaf area) modulates photosynthesis – nitrogen relations: evidence from within and across species and functional groups. Functional Ecology 12, 948–958.

[CIT0033] Reich PB Walters MB Ellsworth DS 1997 From tropics to tundra: global convergence in plant functioning. Proceedings of the National Academy of Sciences, USA 94, 13730–13734. 10.1073/pnas.94.25.13730 PMC28374 9391094

[CIT0034] Roderick ML Berry SL Noble IR Farquhar GD 1999a A theoretical approach to linking the composition and morphology with the function of leaves. Functional Ecology 13, 683–695.

[CIT0035] Roderick ML Berry SL Saunders AR Noble IR 1999b On the relationship between the composition, morphology and function of leaves. Functional Ecology 13, 696–710.

[CIT0036] Sack L Holbrook NM 2006 Leaf hydraulics. Annual Review of Plant Biology 57, 361–381. 10.1146/annurev.arplant.56.032604.144141 16669766

[CIT0037] Sack L Scoffoni C 2013 Leaf venation: structure, function, development, evolution, ecology and applications in the past, present and future. New Phytologist 198, 983–1000. 23600478 10.1111/nph.12253

[CIT0038] Sack L Scoffoni C John GP Poorter H Mason CM Mendez-Alonzo R Donovan LA 2013 How do leaf veins influence the worldwide leaf economic spectrum? Review and synthesis. Journal of Experimental Botany 64, 4053–4080. 24123455 10.1093/jxb/ert316

[CIT0039] Sagan C 1980 Encyclopedia Galactica. Cosmos: A Personal Voyage , *Episode 12.*

[CIT0040] Small E 1972 Photosynthetic rates in relation to nitrogen recycling as an adaptation to nutrient deficiency in peat bog plants. Canadian Journal of Botany 50, 2227–2233.

[CIT0041] Tcherkez G Boex-Fontvieille E Mahe A Hodges M 2012 Respiratory carbon fluxes in leaves. Current Opinion in Plant Biology 15, 308–314. 22244081 10.1016/j.pbi.2011.12.003

[CIT0042] Terashima I Hanba YT Tholen D Niinemets U 2011 Leaf functional anatomy in relation to photosynthesis. Plant Physiology 155, 108–116. 21075960 10.1104/pp.110.165472 PMC3075775

[CIT0043] Tomás M Flexas J Copolovici L Galmés J Hallik L Medrano H Ribas-Carbó M Tosens T Vislap V Niinemets Ü 2013 Importance of leaf anatomy in determining mesophyll diffusion conductance to CO_2_ across species: quantitative limitations and scaling up by models. Journal of Experimental Botany 64, 2269–2281. 23564954 10.1093/jxb/ert086 PMC3654418

[CIT0044] Tosens T Niinemets U Westoby M Wright IJ 2012 Anatomical basis of variation in mesophyll resistance in eastern Australian sclerophylls: news of a long and winding path. Journal of Experimental Botany 63, 5105–5119. 22888123 10.1093/jxb/ers171 PMC3430992

[CIT0045] Villar R Ruiz-Robleto J Ubera JL Poorter H 2013 Exploring variation in leaf mass per area (LMA) from leaf to cell: an anatomical analysis of 26 woody species. American Journal of Botany 100, 1969–1980. 24107583 10.3732/ajb.1200562

[CIT0046] Villar R Robleto JR De Jong Y Poorter H 2006 Differences in construction costs and chemical composition between deciduous and evergreen woody species are small as compared to differences among families. Plant, Cell and Environment 29, 1629–1643. 10.1111/j.1365-3040.2006.01540.x 16898023

[CIT0047] Waite M Sack L 2010 How does moss photosynthesis relate to leaf and canopy structure? Trait relationships for 10 Hawaiian species of contrasting light habitats. New Phytologist 185, 156–172. 19863726 10.1111/j.1469-8137.2009.03061.x

[CIT0048] Witkowski ETF Lamont BB 1991 Leaf specific mass confounds leaf density and thickness. Oecologia 88, 486–493. 10.1007/BF00317710 28312617

[CIT0049] Wright IJ Reich PB Cornelissen JHC 2005 Modulation of leaf economic traits and trait relationships by climate. Global Ecology and Biogeography 14, 411–421.

[CIT0050] Wright IJ Reich PB Westoby M 2004 The worldwide leaf economics spectrum. Nature 428, 821–827. 15103368 10.1038/nature02403

[CIT0051] Zwieniecki MA Boyce CK 2014 Evolution of a unique anatomical precision in angiosperm leaf venation lifts constraints on vascular plant ecology. Proceedings of the Royal Society B: Biological Sciences 281 10.1098/rspb.2013.2829 10.1098/rspb.2013.2829 PMC3924076 24478301

